# Supplementary material for: An image-guided microfluidic system for single-cell lineage tracking
Source: PLoS One. 2023 Aug 1;18(8):e0288655. doi: 10.1371/journal.pone.0288655 (PMC10393162; doi:10.1371/journal.pone.0288655)
Supplement: Attachment — Submitted filename: response_to_reviewers_3.docx [file pone.0288655.s002.docx]

Journal Requirements:

When submitting your revision, we need you to address these additional requirements.

1. Please ensure that your manuscript meets PLOS ONE's style requirements, including those for file naming. The PLOS ONE style templates can be found at 
<https://journals.plos.org/plosone/s/file?id=wjVg/PLOSOne_formatting_sample_main_body.pdf> and 
<https://journals.plos.org/plosone/s/file?id=ba62/PLOSOne_formatting_sample_title_authors_affiliations.pdf>

Done

2. Thank you for stating the following financial disclosure: 
   "This work was supported by funding from the French agency ANR (SinCity; ANR-17-CE12-0031)."

Please state what role the funders took in the study.  If the funders had no role, please state: "The funders had no role in study design, data collection and analysis, decision to publish, or preparation of the manuscript." 
If this statement is not correct you must amend it as needed. 
Please include this amended Role of Funder statement in your cover letter; we will change the online submission form on your behalf.

Done

3. Thank you for stating the following in your Competing Interests section:  
    "NO"

Please complete your Competing Interests on the online submission form to state any Competing Interests. If you have no competing interests, please state "The authors have declared that no competing interests exist.", as detailed online in our guide for authors at <http://journals.plos.org/plosone/s/submit-now> 
 This information should be included in your cover letter; we will change the online submission form on your behalf.

Done

4. We note that you have stated that you will provide repository information for your data at acceptance. Should your manuscript be accepted for publication, we will hold it until you provide the relevant accession numbers or DOIs necessary to access your data. If you wish to make changes to your Data Availability statement, please describe these changes in your cover letter and we will update your Data Availability statement to reflect the information you provide.

Done

5. Please include your full ethics statement in the 'Methods' section of your manuscript file. In your statement, please include the full name of the IRB or ethics committee who approved or waived your study, as well as whether or not you obtained informed written or verbal consent. If consent was waived for your study, please include this information in your statement as well.

Not applicable (no human subjects involved).

Review Comments to the Author

Reviewer #1: In this paper, the authors have developed a multilayer microfluidic device and an experimental workflow for tracking non-adherent cell divisions at the single-cell level. The microfluidic platform could trap single-cells in eight independently controlled proliferation chambers, isolate sister cells after division and extract them for downstream analysis. They demonstrated that the platform could track cells over at least two generations. In comparison with related reports, the design is original and novel and the single-cell extraction volume is down to 500 nL. However, there are typos in the context need to be corrected like on page 3, line 42 a period is missing and so on.

This typo has been corrected.

Reviewer #2: This paper present a setup of microfluidic system for monitoring cell division and isolation of sister cells for post process such as single cell sequencing. The technique looks fine in general. Some revisions or clarifications are needed before publication for better reproducibility.

1. After division, the sister cells are close to each other even after dissociation. The author mentioned the sister cells were to separate into two different chambers. Please give detailed operation process on how to move one cell into another chamber while keep the other cell stay, as demonstrated in Fig. 3. Wouldn't both of the cells move with the flow to new places?

To answer this comment the following modifications were made in the manuscript:

The following statement was added line 261-274:

The sister cells, resulting from the first division of the mother cell, were separated using Accutase as described above and were moved towards the separation zone which incorporates two control valves (**Fig 1B**). The fluid flow was precisely controlled with the pressure pump by applying a pressure of approximately 10 mbars. Accordingly, sister cells moved into a long serpentine channel that connects cell chambers to the separation area in a controlled manner. When the cells were flowing through the serpentine channel to the separation area a sufficient cell-to-cell distance or spacing (a few hundreds of microns) always occurred. In the separation area, actuation of one of the control valves ensured that one of the cells was driven towards relocation area (or extraction area), while the other cell remained trapped (**S1 video)**. The feedback channel allowed relocation of sister cells after division from the separation zone into the cell trapping chambers. Sister cells separated after division circulated through the feedback channel upon actuation of the control valves and fine control of flow pressure, and were subsequently placed in individual trapping chambers (**Fig 3)**.

We also added supplementary video 1 showing sister separation in the separation area for relocation or extraction. The following statement was added line 570:

**S1** : Video of sister cells exiting the chambers area and moving toward the separation area.

2. Single cells were extracted with capillary glass for sequencing. How was the capillary glass inserted into the well? Through PDMS slab or through any hole punched in PDMS? I don't find description on this part. Directly plugging a capillary glass through PDMS sounds quite difficult, especially into a micron sized well precisely. However, if a hole was pre-punched, wouldn't the fluid flow out due to the driven pressure? Please describe in details for clarification.

To answer this comment the following modifications were made in the manuscript:

The selection paragraph was rewritten as follows (line 290-306):

Each selected cell was delivered to the pre-punched extraction well by applying 10 mbar of pressure from the medium inlet, resulting in a cellular velocity of 10 µm/s, with fluorescence imaging being used to track single-cells after their delivery into the extraction well. Next, single-cells were extracted from the device using a thin graduated capillary tube (**Fig 4A**). The glass capillary tube was inserted into the well to extract the cell via capillarity. The extraction area includes two independently addressable, 1 mm diameter and 3 mm depth open wells for the collection of sister cells (Fig 1C). Cells are led to the pre-punched extraction wells, then, the capillary glass is plugged in the well. Since the tip diameter of the capillary glass is smaller than the well diameter, the capillary glass can be easily introduced into the well. As aforementioned, the driven pressure is very low (~10 mbars) which results in a cell velocity of 10 µm/s allowing an easy tracking of the cell inside the extraction well. After a single cell was driven inside the well, fluid flow was stopped during the extraction process and the single cell was collected via the capillary flow. Therefore, the fluid, included the single cell, did not flow out of the well. Additionally, the well has a reservoir volume of 2.5 µL significantly larger than the extraction volume and thus the fluid containing a single cell would not flow out during the extraction process.
